# Effect of Age and Refractive Error on Local and Global Visual Perception in Chinese Children and Adolescents

**DOI:** 10.3389/fnhum.2022.740003

**Published:** 2022-01-28

**Authors:** Jiahe Gan, Ningli Wang, Shiming Li, Bo Wang, Mengtian Kang, Shifei Wei, Jiyuan Guo, Luoru Liu, He Li

**Affiliations:** ^1^Beijing Tongren Hospital, Capital Medical University, Beijing, China; ^2^State Key Laboratory of Brain and Cognitive Science, Institute of Biophysics, Chinese Academy of Sciences, Beijing, China; ^3^Anyang Eye Hospital, Anyang, China

**Keywords:** local visual perception, global visual perception, myopia, children, age

## Abstract

**Purpose:**

This study investigated the impact of age and myopia on visual form perception among Chinese school-age children.

**Methods:**

This cross-sectional study included 1,074 students with a mean age of 12.1 ± 4.7 (range = 7.3–18.9) years. The mean spherical equivalence refraction (SER) of the participants was −1.45 ± 2.07 D. All participants underwent distance visual acuity (VA), refraction measurement and local and global visual form perception test including orientation, parallelism, collinearity, holes and color discrimination tasks.

**Results:**

The reaction times of emmetropes were slower than those of myopic and high myopic groups on both local (orientation, parallelism, and collinearity) and global discrimination tasks (all *p* < 0.05). A reduction in reaction times was found with increasing age on both local and global discrimination tasks (all *p* < 0.05). Age was significantly associated with both local and global visual perception performance after adjusting for gender, visual acuity and SER (orientation, β = −0.54, *p* < 0.001; parallelism, β = −0.365, *p* < 0.001; collinearity, β = −0.28, *p* < 0.001; holes, β = −0.319, *p* < 0.001; color, β = −0.346, *p* < 0.001).

**Conclusions:**

This study revealed that both local and global visual perception improve with age among Chinese children and that myopes seem to have better visual perception than emmetropes.

## Introduction

The alarming increase of myopia has become a public health concern among children and adolescents, especially in East Asia, (Dolgin, 2015; Holden et al., 2016) and approximately 20% of high school students are expected to exhibit high myopia (defined as < −6 dioptres) by 2050 (Wei et al., 2018).

Apart from axial elongation, optical coherence tomography revealed that myopic eyes were accompanied by decreased retinal thickness and reduced macular volume, which might result in altered visual sensation and some visual perception tasks, especially in high myopes (Kuo et al., 2012, 2018). In contrast, previous studies have indicated that myopia is associated with better intelligence and academic performance, (Teasdale et al., 1988; Saw et al., 2007) so it is hypothesized that individuals who are near-sighted have an advantage in visual perception tasks. However, whether myopic children have altered visual perception of local and global patterns remains unclear.

To investigate local and global visual perception, precise definitions of each are required. Global visual perception was traditionally described by Gestalt theory (Pomerantz, 2003) and later elucidated by Chen et al., who claimed that the global nature of visual perception can be referred to as topological invariants and that our visual system perceives global topological information first rather than local features (Chen, 1982, 2005; Wang et al., 2007). The global topological property of an image is a stable geometrical property that remains constant when the shape of the object changes smoothly, and the most important topological property is characterized by the existence of “hole” stimuli. Local visual perception was defined by the perception of distinct levels of geometrical invariants in descending order of structural stability from projective, affine, and then Euclidean features. Previously, Chen (2005) found that adult subjects could discriminate a topological difference with shorter reaction times and higher accuracy than other local geometrical differences. Meng et al. (2019) showed that aging reduced the performance of local visual perception but did not affect global topological perception in individuals 21 to 78 years of age. However, few studies have provided information regarding the development of local and global visual perception before adulthood. The visual system are still developing in children (Parrish et al., 2005; Barsingerhorn et al., 2018). Previous studies have shown not only the impact of age on ocular biometric parameters during childhood (Li et al., 2013, 2016; Zhu et al., 2013) but also the development of numerous aspects of visual function (Gordon and Mcculloch, 1999; Lewis and Maurer, 2010), such as hyperacuity (Buckingham and Kelly, 1996), stereoacuity (Fox et al., 1986), and contrast sensitivity (Almoqbel et al., 2017; Pei et al., 2017). Therefore, we propose the hypothesis that local and global visual perception will develop and improve with age in school-age children.

In this study, we performed a cross-sectional school-based study in a middle-income Chinese city, Anyang, to investigate (1) whether local and global visual perception varies along with refractive status and (2) the impact of age on local and global visual perception in children 7 to 18 years of age.

## Materials and Methods

### Study Design

This cross-sectional study was an additional study of the Anyang Childhood Eye study (ACES) (Li et al., 2013) designed to explore the visual perception and its associated visual factors of Chinese students living in the Anyang urban area, Henan Province, Central China. The survey was approved by the Ethics Committee of Beijing Tongren Hospital, Capital Medical University. All procedures adhered to the tenets of the Declaration of Helsinki. Written informed consent was obtained from at least one parent of each child, and verbal assent was obtained from all children. The examinations were conducted from December 2016 to April 2017. The present study reports the data relevant to visual perception.

### Participants

A random sample of 1,320 children from 3 elementary schools, 2 middle schools and 1 high school were selected, and a random sample of classes for each grade were included. The inclusion criteria were as follows: (1) no history of ocular, psychiatric or neurological diseases; (2) no achromatopsia or achromatopsia, which may affect the visual perception test; (3) sufficient comprehensive skills to understand instructions and cooperate during the visual perceptual task; and (4) no history of antiepileptic medications or drug/alcohol abuse. The exclusion criteria included reaction times of less than 150 ms or over three standard deviations from the mean in each discrimination task (<3%). Among the 1,320 participants, 1,074 subjects (585 males, 489 females) from the age of 7–18 years were enrolled for final analysis. Participants were statistically calculated based on age and refractive error. Three refractive groups (emmetropia, mild myopia, and moderate and high myopia) and four stratified age groups (7∼9 years, 10∼12 years, 13∼15 years and 16∼18 years) were categorized. There were 445, 424, and 205 participants in the emmetropia, mild myopia, and moderate and high myopia groups, respectively. The numbers of participants in the 7∼9-year, 10∼12-year, 13∼15-year and 16∼18-year age groups were 381, 341, 148 and 233, respectively.

### Ocular Examinations

Distance VA was measured separately for each eye using a logarithm of the minimum angle of resolution (logMAR) VA chart (Precision Vision, La Salle, IL, United States) at a distance of 4 m. Students were tested monocularly with the right eye followed by the left eye. Students were notified in advance to bring their glasses when they visited the ophthalmic examination site and were asked to wear corrected spectacles for the VA examination. Refraction was assessed in a non-cycloplegic state using streak retinoscopy performed by an experienced pediatric optometrist or using the autorefractor (HRK7000 A, Huvitz, Gunpo, South Korea) if subjects could not cooperate with the streak retinoscopy.

### Definitions

The spherical equivalent refractive error (SER) was calculated using the standard formula of the algebraic sum of the dioptric powers of the sphere and half of the cylinder (sphere+0.5*cylinder). Refractive statuses were defined based on the following SER: emmetropia, [−0.50 D, 0.50 D); mild myopia, (−3.00 D, −0.50 D); moderate and high myopia, ≤−3.00 D.

### Stimuli and Procedure

**FIGURE 1:**
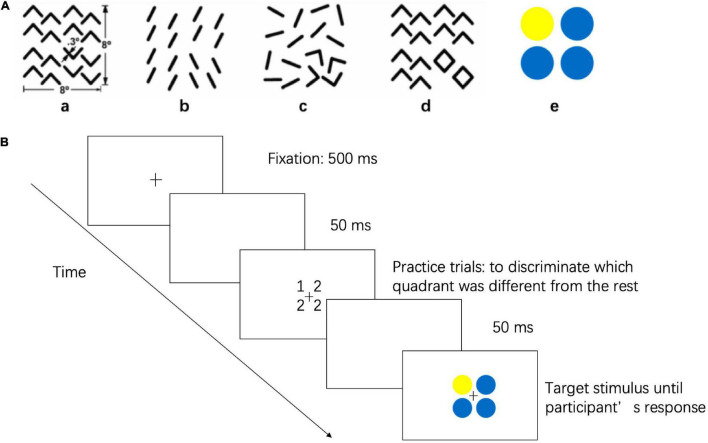
Stimuli and Procedures of Visual Perception Task. **(A)** The stimulus represent discriminations based on **(a)** a difference in angle orientation, a kind of Euclidean property, **(b)** a difference in parallelism, a kind of affine property, **(c)** a difference in collinearity, a kind of projective property, **(d)** a difference in holes, a kind of topological property, and **(e)** a difference in color, a baseline stimulus. **(B)** Flow diagram for visual perception task.

The visual perception task (Chen, 2005) was generated by MATLAB with the Psychophysics Toolbox (Math-Works, Natick, MA, United States) installed on a Lenovo computer (A6800R), and stimuli (8° × 8°) were presented on a 19-inch screen (1,024 pixel × 768 pixel resolution, 100 Hz refresh rate) at an average viewing distance of 58 cm. Subjects were assigned to finish an odd quadrant task, in which they were asked to report which quadrant differed from the other three, as illustrated in Figure 1. The odd quadrant task consisted of five stimulus arrays. Figures 1a–c represent local geometrical discrimination tasks, and Figure 1d represents the global topological discrimination task (Wang et al., 2007). Figure 1a used texture discrimination based on the difference in the orientation, whereas Figure 1b represents a discrimination task based on the difference in parallelism, which is a type of affine property. Figure 1c represents the discrimination task based on the difference in collinearity, which is a type of projective property. Figure 1d was designed to represent a discrimination task based on holes. In Figure 1e, the odd quadrant contains a different color than the other three quadrants, and this quadrant was used to establish a baseline because it was the easiest task.

Each trial started with a 500 ms fixation cross (0.45° × 0.45°) followed by an empty screen for 50 ms. A target stimulus was then shown on the screen until the subject responded. Subjects were asked to maintain eye fixation on the cross throughout the trial. Subjects were instructed to discriminate which quadrant was different from the other three by pressing the correspondence button on a four-button pad, and subjects were asked to reply as accurately and quickly as possible. The five stimuli were presented in a random order, and each stimulus was presented 8 times. The tasks were completed binocularly, and all the myopic subjects were required to wear their spectacles during the test. The test began with five practice trials to ensure that subjects understood the instructions. The mean reaction times and accuracy of the visual perception task were directly extracted from MATLAB software. To correct for the potential speed-accuracy trade-offs, the inverse efficiency score (IE score = mean reaction times/accuracy), which is a standard method of combining reaction times and accuracy into a single performance measure, was used for analysis (Bruyer and Brysbaert, 2011).

### Statistical Analysis

SPSS Statistics version 23 (IBM Corp., Armonk, NY, United States) was used for data analysis, and values are reported as the mean (standard deviation) for continuous variables and percentages for categorical variables. Data from only right eyes were analyzed given that refraction and ocular biometry in the right and left eyes were strongly correlated. Characteristics among the three refractive groups and the four age groups were compared using one-way ANOVAs for continuous variables and χ^2^ tests for categorical variables. The mean reaction times and accuracy of the visual perception task were directly extracted from MATLAB software. Differences in reaction times, accuracy and IE scores for five discrimination tasks among the refractive groups and the age groups were compared using the Kruskal–Wallis *H* test and *post hoc* analysis. Spearman’s partial correlation coefficient was also used to calculate the association between SER and IE scores as well as age and IE scores. Multiple linear regression analysis was used to evaluate the potential association between gender, age, SER, or VA and the reaction times on five discrimination tasks. A *p* value of less than 0.05 was considered statistically significant.

## Results

Among the 1,320 participants, 1,074 (97.0%) subjects with a mean age of 12.1 ± 4.7 (range = 7.3–18.9) underwent a visual perception test and were enrolled for final analysis. Table 1 presents the age, sex, VA and SER of the 1,074 participants according to age and type of refractive error. No significant differences in sex or VA were noted between refractive groups. Age was progressively older as SER decreased in the three refractive groups (10.49 ± 5.80 years vs. 12.24 ± 3.24 years vs. 15.42 ± 2.88 years, respectively; *p* < 0.001), and a myopic shift of SER was identified as age increased in the four stratified age groups (−0.35 ± 0.89 D vs. −1.18 ± 1.65 D vs. −2.10 ± 2.20 D vs. −3.36 ± 2.49 D, respectively; *p* < 0.001).

**TABLE 1:** The characteristics of included populations by type of refractive error and age.

Characteristic (Mean ± SD)	*n*	Age (years)	Gender (No. of male; %)	VA (LogMAR)	Refraction (Diopter)
**Refractive errors**					
Emmetropes	445	10.49 ± 5.80	239(54.3%)	0.06 ± 0.09	0.13 ± 0.45
Mild myopes	424	12.24 ± 3.24	244(57.9%)	0.08 ± 0.17	−1.40 ± 0.82
Moderate and high myopes	205	15.42 ± 2.88	102(50.5%)	0.09 ± 0.16	−5.04 ± 1.53
*P*		<0.001	0.061	0.05	<0.001
**Age**					
7–9	381	8.33 ± 0.41	166(43.6%)	0.06 ± 0.12	−0.35 ± 0.89
10–12	341	11.31 ± 0.86	148(43.4%)	0.09 ± 0.16	−1.18 ± 1.65
13–15	148	14.60 ± 0.88	62(41.9%)	0.07 ± 0.18	−2.10 ± 2.20
16–18	233	17.31 ± 0.94	107(45.9%)	0.07 ± 0.16	−3.36 ± 2.49
*P*		<0.001	0.072	0.09	<0.001

*VA, visual acuity.*

**FIGURE 2:**
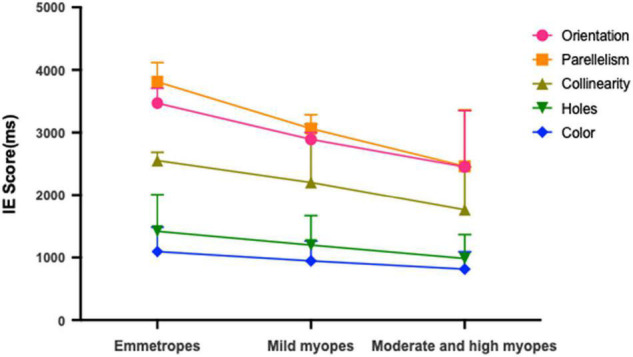
Mean IE scores for emmetropes, mild myopes and moderate and high myopes in global topological (hole), local geometrical (orientation; parallelism; collinearity) and color discrimination tasks.

Table 2 presents the means and standard deviations (SDs) of reaction times for the orientation, parallelism, collinearity, holes and color discrimination tasks between the various refractive groups. For each stimulus type, comparison analyses were performed between refractive groups. Participants with moderate and high myopia had shorter reaction times compared with that of the mild myopia group and emmetropia group in both the local geometrical discrimination tasks and global topological task, which were all statistically significant: [Orientation: moderate and high myopes vs. emmetropes, H_(21,017)_ = 66.556, *p* < 0.001; moderate and high myopes vs. mild myopes, H_(21,017)_ = 17.591, *p* < 0.001; Parallelism: moderate and high myopes vs. emmetropes, H_(2,997)_ = 61.967, *p* < 0.001; moderate and high myopes vs. mild myopes, H_(2,997)_ = 17.619, *p* < 0.001; Collinearity: moderate and high myopes vs. emmetropes, H_(21,014)_ = 64.491, *p* < 0.001; moderate and high myopes vs. mild myopes, H_(21,014)_ = 27.291, *p* < 0.001; Holes: moderate and high myopes vs. emmetropes, H_(21,065)_ = 91.409, *p* < 0.001; moderate and high myopes vs. mild myopes, H_(21,065)_ = 34.409, *p* < 0.001; Color: moderate and high myopes vs. emmetropes, H_(21,013)_ = 65.980, *p* < 0.001; moderate and high myopes vs. mild myopes, H_(21,013)_ = 26.691, *p* < 0.001]. And the mild myopia group showed shorter reaction times than that of the emmetropia group in both the local geometrical discrimination tasks and global topological task [Orientation: mild myopes vs. emmetropes, H_(21,017)_ = 26.843, *p* < 0.001; Parallelism: mild myopes vs. emmetropes, H_(2,997)_ = 19.324, *p* < 0.001; Collinearity: mild myopes vs. emmetropes, H_(21,014)_ = 14.081, *p* < 0.001; Holes: mild myopes vs. emmetropes, H_(21,065)_ = 29.711, *p* < 0.001; Color: mild myopes vs. emmetropes, H_(21,013)_ = 30.669, *p* < 0.001]. No difference in accuracy was observed between refractive groups. To correct for the potential speed-accuracy trade-offs, we also performed a partial correlation analysis of the relationship between the IE scores (IE = mean reaction times/accuracy; Figure 2) of visual perception and SER and found a statistically significant correlation between them in all stimulus types after adjusting by age for the parallelism, collinearity, topological and color discrimination tasks (parallelism: r^2^ = 0.161, *p* < 0.001; collinearity, r^2^ = 0.171, *p* < 0.001; holes, r^2^ = 0.182, *p* < 0.001; color, r^2^ = 0.165, *p* < 0.001, respectively), except for the orientation task (orientation: r^2^ = 0.03, *p* = 0.34).

**TABLE 2:** The reaction times (mean ± SD, ms) on local and global discrimination tasks among the refractive groups and the stratified age groups.

	Orientation	Parallelism	Collinearity	Holes	Color
**Refractive error**					
Emmetropes	2,797 ± 888^*ab*^	2,808 ± 826^*ab*^	2,290 ± 889^*ab*^	1,463 ± 713^*ab*^	1,020 ± 292^*ab*^
Mild myopes	2,519 ± 789^b^	2,535 ± 809^b^	2,035 ± 794^b^	1,243 ± 608^b^	912 ± 264^b^
Moderate and high myopes	2,204 ± 602	2,183 ± 646	1,672 ± 661	994 ± 426	790 ± 207
*P*	<0.001	<0.001	<0.001	<0.001	<0.001
**Age (years)**					
7–9	3,138 ± 781^cde^	3,251 ± 713^cde^	2,564 ± 693^cde^	1,661 ± 590^cde^	1,185 ± 251^cde^
10–12	2,442 ± 637^de^	2,483 ± 654^de^	2,046 ± 807^de^	1,176 ± 461^de^	906 ± 211^de^
13–15	2,190 ± 572	2,149 ± 617	1,741 ± 643	955 ± 264	783 ± 189
16–18	2,044 ± 512	1,995 ± 523	1,453 ± 474	845 ± 226	709 ± 159
*P*	<0.001	<0.001	<0.001	<0.001	<0.001

*P values from ANOVA.*

*SER, spherical equivalent in the better-seeing eyes.*

*^a^Compared with myopes, P < 0.001; ^b^Compared with myopes, P < 0.001; ^c^Compared with 10–12 years old group, P < 0.001; ^d^Compared with 13–15 years old group, P < 0.001; ^e^Compared with 16–18 years old group, P < 0.001.*

Separate analyses for each refractive group were performed to explore the effect of SER between the five stimulus types. In the emmetropia group, a significant effect of stimulus type on reaction times was found [F_(42,220)_ = 12.512, *p* < 0.001]. *Post hoc* analysis within the emmetropia group revealed a statistically significant difference between the reaction times of the global discrimination of holes and local geometrical discrimination: orientation vs. holes, H_(42,225)_ = 1185.855, *p* < 0.001; parallelism vs. holes, H_(42,225)_ = 1109.84, *p* < 0.001; collinearity vs. holes, H_(42,225)_ = 549.068, *p* < 0.001. Second, there was a superior effect of collinearity over parallelism [H_(42,225)_ = 156.611, *p* < 0.001] and orientation [H_(42,225)_ = 151.257, *p* < 0.001]. Third, discrimination based on orientation was quantitively but not statistically slower that based on parallelism [H_(42,225)_ = 3.285, *p* = 0.699]. As the baseline of the easiest task, reaction times for color discrimination were remarkably faster than orientation, parallelism and collinearity tasks (all *p*s < 0.001). The same trend of the effect of stimulus types on reaction times was also observed in the mild myopia group. Unlike the emmetropia and mild myopia groups, the orientation discrimination task was quantitively but not statistically faster, than that of the parallelism task [H_(41,019)_ = 13.84, *p* = 0.07] in the moderate and high myopia groups.

**FIGURE 3:**
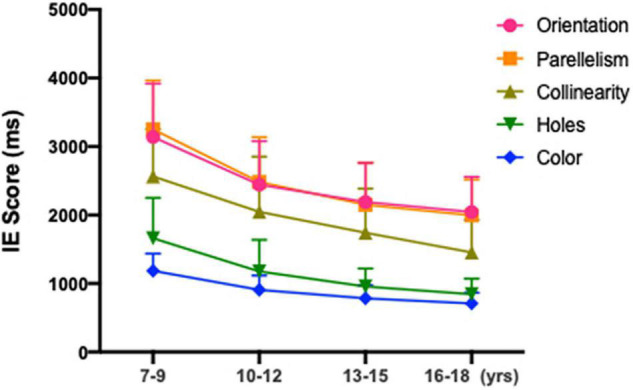
Mean IE scores for subjects aged 7–9, 10–12, 13–15, and 16–18 years old in global topological (hole), local geometrical (orientation; parallelism; collinearity) and color discrimination tasks.

*Post hoc* analyses were performed to compare the differences between age groups (as illustrated in Table 2). The older group of 16–18 years showed a shorter reaction times in both the local geometrical discrimination tasks and global topological task with that of the age groups of 7–9, 10–12 years [Orientation: 16–18 vs. 7–9, H_(21,005)_ = 194.542, *p* < 0.001; 16–18 vs. 10–12, H_(21,005)_ = 45.402, *p* < 0.001; Parallelism: 16–18 vs. 7–9, H_(2,986)_ = 225.086, *p* < 0.001; 16–18 vs. 10–12, H_(2,986)_ = 62.129, *p* < 0.001; Collinearity: 16–18 vs. 7–9, H_(21,003)_ = 455.838, *p* < 0.001; 16–18 vs. 10–12, H_(21,003)_ = 236.445, *p* < 0.001; Holes: 16–18 vs. 7–9, H_(21,052)_ = 26.201, *p* < 0.001; 16–18 vs. 10–12, H_(21,052)_ = 25.913, *p* < 0.001; Color: 16–18 vs. 7–9, H_(21,000)_ = 25.430, *p* < 0.001; 16–18 vs. 10–12, H_(21,000)_ = 25.037, *p* < 0.001], whereas no significant difference was found between 13–15 years of age and 16–18 years of age (all *p* > 0.05). No significant differences in accuracy were noted among the four age groups. As shown in Figure 3, partial correlation analysis showed that the relationship between the IE scores for all stimulus types was still significant after adjusting for SER (orientation, r^2^ = −0.443, *p* < 0.001; parallelism, r^2^ = −0.349, *p* < 0.001; collinearity, r^2^ = −0.299, *p* < 0.001; holes, r^2^ = −0.327, *p* < 0.001; color, r^2^ = −0.354, *p* < 0.001).

We also conducted separate analyses for each age group to explore the effect of age between the five stimuli. In the 7∼9 age group, a significant effect of stimulus type on reaction times was observed [F_(41,790)_ = 981.765, *p* < 0.001]. *Post hoc* analysis within the 7∼9 age group revealed a statistically significant shorter reaction times of the global discrimination of holes than those in local geometrical discrimination tasks: orientation vs. holes, H_(41,790)_ = 404.255, *p* < 0.001; parallelism vs. holes, H_(41,790)_ = 444.268, *p* < 0.001; collinearity vs. holes, H_(41,790)_ = 225.706, *p* < 0.001. Second, there was a superior effect of collinearity over parallelism [H_(41,790)_ = 117.459, *p* < 0.001] and orientation [H_(41,790)_ = 32.29, *p* < 0.001]. Third, discrimination based on orientation was quantitively but not statistically faster than that based on parallelism [H_(41,790)_ = 16.9, *p* = 0.05]. Reaction times for color discrimination were remarkably faster than orientation, parallelism, collinearity and holes tasks in all age groups (all *p*s < 0.001). The same trend was also found in the 10∼12 age group. Unlike the 7∼9 and 10∼12 age groups, the orientation discrimination task was quantitively but not statistically faster than that based on parallelism in the 13∼15 and 16∼18 age groups.

As shown in Table 3, the multiple linear regression model demonstrated that after adjustment for gender, age and VA, SER significantly contributed to the reaction times for the parallelism, collinearity, topological and color discrimination tasks (parallelism, standardized β = 0.149, *p* < 0.001; collinearity, standardized β = 0.169, *p* < 0.001; holes, standardized β = 0.139, *p* < 0.001; color, standardized β = 0.186, *p* < 0.001) but not the orientation discrimination task (standardized β = −0.033, *p* = 0.307). Age significantly contributed to all the stimulus types (orientation, standardized β = −0.54, *p* < 0.001; parallelism, standardized β = −0.365, *p* < 0.001; collinearity, standardized β = −0.28, *p* < 0.001; holes, standardized β = −0.319, *p* < 0.001; color, standardized β = −0.346, *p* < 0.001) after adjustment for the other observed factors.

**TABLE 3:** Multiple linear regression model of reaction times for local and global discrimination tasks.

	Orientation		Parallelism		Collinearity		Holes		Color	
Variable	Standardized β	*P*	Standardized β	*P*	Standardized β	*P*	Standardized β	*P*	Standardized β	*P*
Gender	0.056	0.051	0.004	0.899	0.004	0.895	0.014	0.638	0.026	0.359
Age	−0.540	**< 0.001**	−0.365	**< 0.001**	−0.280	**< 0.001**	−0.319	< 0.001	−0.346	**< 0.001**
SER	−0.033	0.307	0.149	**< 0.001**	0.169	**< 0.001**	0.139	< 0.001	0.186	**< 0.001**
VA	−0.039	0.161	−0.011	0.720	0.001	0.972	−0.003	0.913	0.021	0.471

*SER, spherical equivalent refractive error; VA, visual acuity.*

*Bold values indicate a statistically significant difference, P < 0.05.*

## Discussion

The current study suggests that higher myopes exhibited better performance, i.e., shorter reaction times, for local and global visual perception than emmetropes. Furthermore, the results indicate that the performance of both local and global visual perception improves with age in school-age children with continuous data from 7 to 18 years of age.

This is the first study to provide the association between visual perception and refractive status in Chinese children and adolescents. The result that higher myopia patients exhibited better performance on local and global visual perception remained evident when adjusting for age. On the one hand, better academic performance and cognitive function were previously noted in myopes (Teasdale et al., 1988; Saw et al., 2007; Czepita et al., 2008; Megreli et al., 2020). Saw et al. (2004) observed a higher non-verbal IQ in myopes, which indicated better cognitive performance. The Gutenberg Health Study (Mirshahi et al., 2016) also reported that a higher cognitive ability was identified in myopes compared with non-myopes with the Tower of London test (TOL) in adults 40–79 years of age, and the TOL score increased with the magnitude of myopia. The study suggested that cognitive ability may be associated with myopia primarily through its impact on educational behavior. Students with myopia are more likely to engage in increased levels of near-work activities (Saw et al., 2001; Li et al., 2015). Therefore, students with a higher degree of myopia may perform better at near work tasks, including local and global visual perception tasks. Furthermore, some researchers suggest that myopia is an overdevelopment of the eye, and as ocular development is reported to be related to neurocognitive development, this in turn leads to superior intelligence among myopes (Mak et al., 2006; Megreli et al., 2020). Another possible explanation is that both myopia and neurocognitive function are affected by the same gene or set of genes (Karlsson, 1975; Williams et al., 2017). On the other hand, the study of Guo (Li et al., 2012) found that the correlation between the visual cortex and visual-related areas is strengthened in patients with high myopia compared with emmetropes. Guo et al. demonstrated that individuals with myopia have almost normal visual acuity after correction with spectacles; thus, their cortices develop normally. With refractive error in highly myopic patients being compensated by the increased connection between the visual cortex and related areas, myopic individuals may have better performance on local and global perception due to an increased ability to collect and integrate visual signals.

However, some studies found that high myopia may be associated with reduced visual function, such as dot motion perception (Kuo et al., 2018), whereas the present study indicates better local and global visual perception in higher myopia. This finding is potentially explained by the fact that local and global visual perception are the primitive function of our cognitive ability, especially global topological perception according to Chen’s “global first” theory (Chen, 2005). Furthermore, poor performance on particular visual perception tasks in high myopes may result from reduced activity of the magnocellular pathway in elongated myopic eyes, but research indicates that global topological perception is processed *via* the subcortical pathway from retina to superior colliculus and then to higher cortex (Roy et al., 2017; White et al., 2017; Meng et al., 2018), and no evidence has supported subcortical pathway impairment in myopic populations thus far.

The findings from the current study also revealed a significant improvement in local and global visual perception with age among Chinese children from 7 to 18 years of age. Our findings complement previous Meng’s findings, who reported age-related perceptual deterioration in local geometrical perception from 21 to 78 years of age (Meng et al., 2019). Therefore, it was found that before adulthood, local and global visual perception continues to improve with age. However, after adulthood, local geometrical perception begins to decline. In contrast, global topological function remains stable during the aging process. The mechanisms underlying age-related changes in visual perception remains unclear. Previous studies have shown substantial maturation of functional brain networks and cognitive abilities (Laurence, 2005; Le et al., 2020), including working memory (Shing et al., 2010) and cognitive flexibility (Dajani and Uddin, 2015), during childhood and adolescence. Recent neuroimaging research has also suggested that the visual system matures at different levels in line with the increasing complexity within the visual system (Klaver et al., 2011). These researchers suggested that visual functions mature until late childhood. Research has demonstrated that visual acuity improved with age, most dramatically in the first 24 months of life, followed by a slower improvement continuing up to 72 months and likely beyond (Leone et al., 2014). Other aspects of visual function, such as contrast sensitivity (Leat et al., 2009), hyperacuity (Buckingham and Kelly, 1996) and stereoacuity (Fox et al., 1986), continue to develop until 8–12 years of age.

The results shown in Table 2 also demonstrate a correlation between reaction times and different levels of geometrical invariants stratified by Klein’s Program among three refractive groups and four age groups. In particular, except for the baseline color task, global topological perception occurred prior to the perception of other local geometrical properties among three refractive groups and four age groups. These behavioral data could be explained by the “global precedence hypothesis” firstly put forward by Navon in 1977 and further elucidated by Chen Lin as “early topological perception,” in which he pointed out that a correlation between reaction times and the stratification of the geometric configuration (Chen, 2005). Interestingly, we found that in the moderate and high myopia group, 13–15 year-old group and the 16–18 year-old group, the reaction times of parallelism were not statistically significant, but qualitatively faster than that of the orientation discrimination task, which were consistent with previous research in adults (Meng et al., 2019). However, the reaction times of parallelism were found to be qualitatively slower than that of orientation in the 7–9 year-old group and the 10–12 year-old group, as well as the emmetropia and mild myopia groups. Though the difference of reaction times between these two tasks did not reach statistical significance, these may indicate the development of visual form perception until early teenagers, since subjects in the emmetropia and mild myopia groups are younger than those in the moderate and high myopia group.

The strengths of the present study include a relatively large sample size and the adjustment for potential confounders. In addition, this is the first cross-sectional study investigating the association between visual perception and age as well as refractive status in school children. However, our study also had limitations. First, the visual perceptual test applied in the current study was a behavioral test using psychophysical techniques rather than objective techniques. The conclusion may be specific to the stimuli and tasks used. Second, the variances among the four age groups may result from the different way that subjects may perform in a testing situation. For example, younger children may have more variable or shorter attention or are more likely to be influenced by cognitive fatigue (Sievertsen et al., 2016). Third, visual perception relies on both optical and neural factors. Therefore, the correlation between visual perception and cognitive functions (Cui et al., 2019) is worth further exploration in the future.

## Conclusion

In conclusion, the study revealed that both global and local visual perception performance improved with ag, and myopes exhibited better performance on local and global visual perception than emmetropes.

## Data Availability Statement

The raw data supporting the conclusions of this article will be made available by the authors, without undue reservation.

## Ethics Statement

The studies involving human participants were reviewed and approved by the Institutional Review Board of Beijing Tongren Hospital, Capital Medical University. Written informed consent to participate in this study was provided by the participants’ legal guardian/next of kin.

## Author Contributions

JGa, NW, SL, MK, SW, BW, JGu, and LL conceived the experiments. JGa, MK, SW, JGu, and LL performed the experiments. JGa, BW, SL, and NW analyzed and interpreted the data and wrote the manuscript. All authors contributed to manuscript revision, read, and approved the submitted version.

## Conflict of Interest

The authors declare that the research was conducted in the absence of any commercial or financial relationships that could be construed as a potential conflict of interest. The handling editor declared a shared parent affiliation with several of the authors, JGa, NW, SL, MK, and SW at the time of review.

## Publisher’s Note

All claims expressed in this article are solely those of the authors and do not necessarily represent those of their affiliated organizations, or those of the publisher, the editors and the reviewers. Any product that may be evaluated in this article, or claim that may be made by its manufacturer, is not guaranteed or endorsed by the publisher.
